# Climate and Soil Constraints Shape Vegetation-Zone-Dependent Leaf C:N:P Stoichiometry of *Quercus variabilis*

**DOI:** 10.3390/plants15142219

**Published:** 2026-07-21

**Authors:** Yanqi Yuan, Kaixi Chen, Xue Wang, Xinrui Liu, Zemeng Ma, Shuoxin Zhang, Fan Hao, Zhouping Shangguan

**Affiliations:** 1 State Key Laboratory of Soil and Water Conservation and Desertification Control, The Research Center of Soil and Water Conservation and Ecological Environment, Chinese Academy of Sciences and Ministry of Education, Yangling 712100, China; yuanyanqi123@nwafu.edu.cn; 2 Institute of Soil and Water Conservation, Chinese Academy of Sciences and Ministry of Water Resources, Yangling 712100, China; 3 University of Chinese Academy of Sciences, Beijing 100049, China; 4 College of Forestry, Northwest A&F University, Yangling 712100, China; kxchen@nwafu.edu.cn (K.C.); mazemeng@nwafu.edu.cn (Z.M.); sxzhang@nwsuaf.edu.cn (S.Z.); 5 Inner Mongolia Academy of Forestry Sciences, Hohhot 010010, China; wangxue202509@163.com; 6 College of Forestry, Inner Mongolia Agriculture University, Hohhot 010018, China; xinruiliu@nwafu.edu.cn

**Keywords:** *Quercus variabilis*, leaf C:N:P stoichiometry, between-zone differentiation, environmental factors, vegetation zones

## Abstract

Leaf C:N:P stoichiometry provides a functional link between plant nutrient composition and environmental adaptation. However, whether widely distributed tree species maintain consistent leaf stoichiometric patterns across contrasting vegetation zones remains unclear. Here, we investigated the leaf C:N:P stoichiometry of *Quercus variabilis*, a widely distributed tree species in China, across the subtropical evergreen broad-leaved forest zone (SEF) and the warm-temperate deciduous broad-leaved forest zone (WDF). We aimed to clarify between-zone differences in leaf stoichiometric traits and the role of climate and soil constraints in shaping vegetation-zone-dependent stoichiometric variation. Our results showed that leaf C:N:P stoichiometry of *Q. variabilis* differed between the SEF and WDF, with significant differences detected only for leaf N and leaf C:N. Overall, leaf C varied little across zones (CV = 3.13–3.42%), leaf N and leaf C:N showed moderate variation (CV = 10.07–13.26%), whereas leaf P, leaf C:P, and leaf N:P showed greater variability (CV = 33.28–39.93%). Environmental analyses further indicated contrasting patterns between zones: leaf stoichiometry in the SEF was jointly associated with climate and soil factors and showed a positive leaf C–N relationship, whereas that in the WDF showed a stronger soil association and a negative leaf N–P relationship. These findings suggest that *Q. variabilis* may adjust its leaf stoichiometry in a zone-dependent manner in response to contrasting climate and soil conditions. This study provides a theoretical basis for understanding nutrient-use strategies and environmental adaptation of widely distributed tree species under heterogeneous climate and soil conditions.

## 1. Introduction

Variation in plant functional traits along environmental gradients provides a fundamental basis for understanding species’ ecological adaptation and the maintenance of ecosystem functioning. As a central component of ecological stoichiometry, leaf C:N:P stoichiometry reflects plant nutrient-use strategies and responses to environmental change [1,2]. Carbon contributes to both energy supply and leaf structural construction, nitrogen is a major constituent of proteins and enzymes involved in physiological processes, and phosphorus plays essential roles in nucleic acid synthesis, energy transfer, and cell division; together, these elements regulate plant growth [1,3]. Therefore, leaf C, N, and P concentrations and their ratios not only reflect trade-offs among resource acquisition, structural investment, and metabolic maintenance, but also are closely linked to community assembly, ecosystem nutrient cycling, and plant responses to environmental change [1,4,5,6]. Previous studies have shown that leaf N, P, and N:P exhibit pronounced spatial patterns at both global and China-wide scales, and are closely associated with climate, latitude, and soil conditions [7,8,9]. However, because current understanding is largely derived from multi-species or community-level studies, it remains unclear whether a single widely distributed tree species maintains a consistent leaf stoichiometric pattern across different vegetation zones.

Unlike multi-species comparisons, studies focusing on a single tree species can partly reduce the confounding effects of phylogeny and species turnover, thereby providing a more direct assessment of how environmental context shapes intraspecific stoichiometric variation [10,11]. Previous studies have shown that leaf N, P, and N:P can vary with geographic and climate conditions among *Quercus* species, and that their geographic patterns may be more complex than those explained by individual climate variables alone [12]. These findings suggest that widely distributed tree species may not maintain fixed leaf stoichiometric patterns, as elemental concentrations and element–element relationships can vary among sites and vegetation zones. However, most existing studies have focused on spatial variation in individual elements, leaving limited understanding of whether relationships among leaf C, N, and P shift across different environmental contexts.

Leaf C:N:P stoichiometry may vary both among vegetation zones and among sites within the same zone. At broad spatial scales, temperature, precipitation, and latitude are often associated with geographic variation in leaf N, P, and N:P ratios [7,8,9,12]. Soil conditions may further contribute to among-site variation in leaf stoichiometry, suggesting that leaf C:N:P patterns are shaped by climate and soil conditions rather than by vegetation zone itself [9].

Thus, geographic gradients can be viewed as spatial proxies for underlying environmental variation rather than as direct regulators of leaf stoichiometry [7,13]. In China, the subtropical evergreen broad-leaved forest zone and the warm-temperate deciduous broad-leaved forest zone differ markedly in temperature, precipitation, seasonality, and soil nutrient backgrounds, providing a suitable context for testing between-zone variation in leaf stoichiometry within a single tree species. Subtropical forests are generally warmer and wetter, where climate may influence leaf stoichiometry through direct effects on plant nutrient demand and indirect effects on soil weathering, mineralization, and nutrient availability [7,14,15,16]. By contrast, warm-temperate forests experience stronger seasonality, under which local soil resource availability may be more closely associated with leaf nutrient concentrations and ratios [7,16]. Together, these contrasts suggest that the same tree species distributed across different vegetation zones may exhibit divergent environmental associations and element–element relationships.

*Quercus variabilis* is a widely distributed deciduous broad-leaved tree species in China, spanning both the subtropical evergreen broad-leaved forest zone (SEF) and the warm-temperate deciduous broad-leaved forest zone (WDF). It therefore provides an ideal model for examining broad-scale environmental adaptation within a single tree species. In this study, climate–soil co-regulation refers to the joint contribution of climate and soil variables to the explained variation in leaf stoichiometry. Elemental coordination and trade-offs refer to positive and negative relationships among leaf elements, respectively; these terms describe statistical associations rather than direct physiological causality. However, it remains poorly understood whether the leaf C:N:P stoichiometry of *Q. variabilis* differs between vegetation zones, and how climate, soil, and element–element relationships are involved in such differences. To address these knowledge gaps, we compared leaf C, N, and P concentrations and their stoichiometric ratios in *Q. variabilis* between the SEF and WDF, and further examined their relationships with geographic gradients, climate, and soil factors. Specifically, we aimed to answer the following questions: (1) whether the leaf stoichiometry of *Q. variabilis* differs significantly between vegetation zones; (2) whether such differences are associated with different relative contributions of climate and soil variables; and (3) whether relationships among leaf elements differ between vegetation zones. We hypothesized that *Q. variabilis* does not maintain a fixed leaf stoichiometric pattern across vegetation zones, but instead shows zone-dependent variation associated with contrasting climate and soil backgrounds. Specifically, we expected climate and soil variables to jointly contribute to leaf stoichiometric variation in the SEF, whereas soil variables would make a stronger relative contribution in the WDF. We further expected positive element–element relationships to be more evident in the SEF, whereas negative relationships among nutrient elements would be more evident in the WDF. By focusing on a single widely distributed tree species and integrating elemental concentrations, environmental factors, and element–element relationships, this study provides evidence for understanding how leaf stoichiometric patterns vary across contrasting vegetation-zone contexts.

## 2. Materials and Methods

### 2.1. Study Area and Leaf Collection

**Figure 1:**
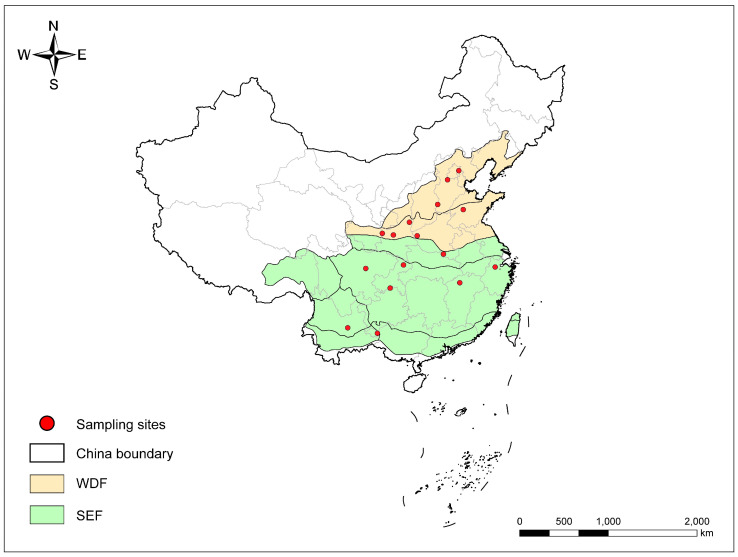
Locations of sampling sites in the SEF and WDF. SEF, subtropical evergreen broad-leaved forest zone; WDF, warm-temperate deciduous broad-leaved forest zone.

We selected *Quercus variabilis*, a widely distributed tree species in China, as the study species. From August to October 2021, eight sampling sites were established in each of the subtropical evergreen broad-leaved forest zone (SEF) and the warm-temperate deciduous broad-leaved forest zone (WDF) for field sampling, resulting in a total of 16 sampling sites (Figure 1). The sampling sites ranged from 24°26′12″ to 40°15′14″ N and from 102°37′06″ to 120°00′25″ E. Across these sites, mean annual temperature (MAT) varied from 6.2 to 21.1 °C, and mean annual precipitation (MAP) ranged from 781 to 2795 mm. Basic information on the sampling sites is provided in Table 1.

At each sampling site, mature natural forests were chosen for the establishment of sample plots. Within each site, three 20 m × 20 m sample plots were established, with a minimum distance of 30 m maintained between adjacent plots. In each plot, mature and healthy leaves were collected from the upper-middle outer canopy using a pole pruner. The collected leaves were then cleaned to remove surface impurities, placed in sealed bags, and stored for subsequent laboratory analysis.

### 2.2. Leaf Measurements

Leaf samples were first heated at 105 °C for 30 min to stop enzymatic activity, and then dried at 70 °C for 48 h to constant mass. The dried samples were ground into a fine powder using a grinder and passed through a 100-mesh sieve for elemental analysis. Leaf carbon (LC) concentration was measured using the potassium dichromate volumetric method [17]. After H_2_SO_4_–H_2_O_2_ digestion, leaf nitrogen (LN) and leaf phosphorus (LP) concentrations were determined using a Kjeltec analyzer (Kjeltec 2300 Analyzer Unit, Foss Tecator, Hoganas, Sweden) and the molybdenum yellow colorimetric method with a U-2800 spectrophotometer (Shanghai, China), respectively [17].

### 2.3. Environmental Data

Mean annual temperature (MAT, °C) and mean annual precipitation (MAP, mm) were obtained from the China Meteorological Data Service Centre. Soil total phosphorus (STP) data were collected from the global soil total phosphorus dataset published by He et al. [18]. Other soil properties, including soil bulk density (SBD), soil pH, soil organic carbon (SOC), and soil total nitrogen (STN), were obtained from the Harmonized World Soil Database (HWSD 2.0). To ensure consistency among variables, all soil properties were extracted for the 0–20 cm soil layer to represent topsoil conditions. The vegetation regionalization map of China was downloaded from the Resource and Environmental Science Data Platform [19]. Environmental variables were extracted for each sampling site and used in subsequent statistical analyses. Soil variables derived from gridded datasets were interpreted as regional topsoil background conditions rather than direct plot-scale measurements.

### 2.4. Statistical Analysis

To compare the overall characteristics and between-zone differences in leaf stoichiometric traits, descriptive statistics, including the mean, standard deviation (SD), minimum (Min), maximum (Max), and coefficient of variation (CV), were calculated for each trait. As most variables did not meet the assumption of normality, the Wilcoxon rank-sum test was used to assess differences between the two vegetation zones. In addition, linear regression analyses were performed to examine changes in leaf stoichiometric traits along geographic gradients, represented by latitude and longitude.

To assess the relative importance of environmental factors in leaf stoichiometric variation, variance partitioning analysis (VPA) was used to quantify the independent and shared contributions of climate and soil variables [20]. Multiple linear regression models were then constructed, and the final models were selected using bidirectional stepwise selection. The relative contribution of each retained predictor was further evaluated using the Lindeman–Merenda–Gold (LMG) metric implemented in the “relaimpo” package [21]. To minimize multicollinearity among explanatory variables, only variables with variance inflation factors (VIFs) < 5 were retained. The final predictor set included five environmental variables: MAT, MAP, soil pH, STN, and STP. This same predictor set was used consistently for VPA, multiple linear regression, and relative importance analysis.

To examine the relationships among stoichiometric traits in the two vegetation zones, Spearman’s rank correlation was first used to assess pairwise associations. Standardized major axis regression (SMA), implemented in the “smatr” package, was then used to quantify allometric relationships among leaf elemental traits [22].

All statistical analyses were conducted using SPSS 31.0.0 (IBM Corp., Armonk, NY, USA) and R 4.4.3 (R Foundation for Statistical Computing, Vienna, Austria).

## 3. Results

### 3.1. Differences in Leaf C:N:P Stoichiometry Between Vegetation Zones

The leaf C:N:P stoichiometry of *Q. variabilis* exhibited trait-specific differences between the two vegetation zones (Table 2). Mean LC, LP, and LC/LN were higher in the SEF, whereas mean LN, LC/LP, and LN/LP were higher in the WDF. However, only LN and LC/LN differed significantly between zones (*p* < 0.05). The magnitude of variation also differed substantially among stoichiometric traits. LC exhibited the lowest variation in both vegetation zones (CV = 3.13–3.42%), indicating relatively conservative LC variation. LN and LC/LN showed moderate variation (CV = 10.07–13.26%). In contrast, LP, LC/LP, and LN/LP varied more strongly (CV = 33.28–39.93%), suggesting greater spatial variability in P-related stoichiometric traits.

### 3.2. Geographic Patterns of Leaf C:N:P Stoichiometry

**Figure 2:**
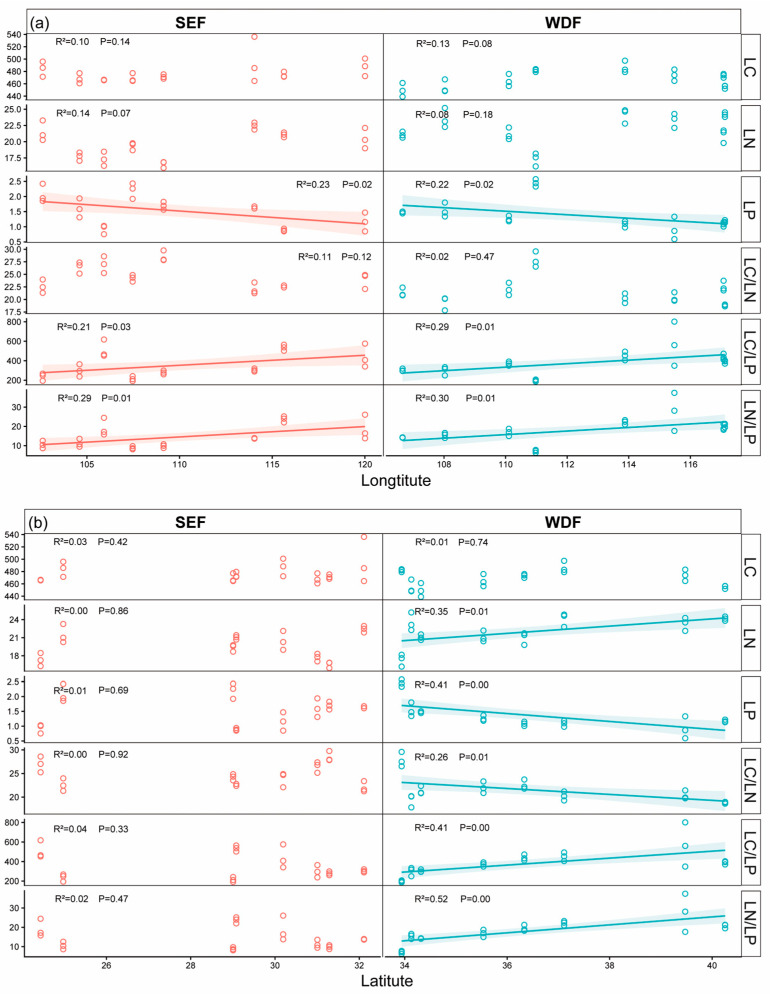
Longitudinal and latitudinal patterns of leaf C:N:P stoichiometric traits across the two vegetation zones. (**a**) Longitudinal gradient; (**b**) latitudinal gradient. Open circles represent sampling-site observations. Solid lines indicate significant linear relationships, and shaded areas represent 95% confidence intervals. Regression lines are shown only for significant relationships. R^2^ and *p* values are given in each panel. SEF, subtropical evergreen broad-leaved forest zone; WDF, warm-temperate deciduous broad-leaved forest zone.

Leaf C:N:P stoichiometry showed distinct geographic patterns between the two vegetation zones (Figure 2). Along the longitudinal gradient, LP decreased significantly with increasing longitude in both the SEF and WDF (*p* < 0.05), whereas LC/LP and LN/LP increased significantly (*p* < 0.05). The remaining traits showed no significant longitudinal trends. Along the latitudinal gradient, no stoichiometric traits showed significant relationships in the SEF. In contrast, in the WDF, LN, LC/LP, and LN/LP increased significantly with increasing latitude (*p* < 0.05), whereas LP and LC/LN decreased significantly (*p* < 0.05). These results indicate that the leaf C:N:P stoichiometry of *Q. variabilis* in the WDF was more closely associated with latitude than that in the SEF.

### 3.3. Relationships Between Leaf C:N:P Stoichiometry and Environmental Factors

**Figure 3:**
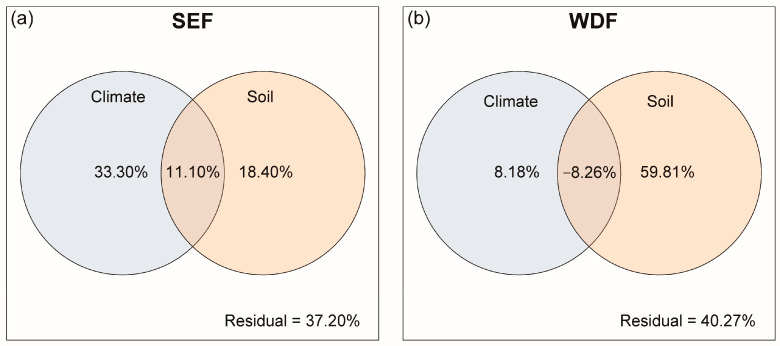
Variance partitioning of climate and soil contributions to leaf C:N:P stoichiometric variation across the two vegetation zones. (**a**) SEF; (**b**) WDF. Values represent adjusted fractions (%) of variation independently or jointly explained by climate and soil variables. “Climate” and “Soil” indicate the independent fractions explained by climate and soil variables, respectively; the overlapping area indicates the fraction jointly explained by both variable groups. “Residual” represents unexplained variation. The negative shared fraction in the WDF resulted from adjusted variance partitioning and was interpreted as negligible rather than as a meaningful negative joint effect. SEF, subtropical evergreen broad-leaved forest zone; WDF, warm-temperate deciduous broad-leaved forest zone.

**Figure 4:**
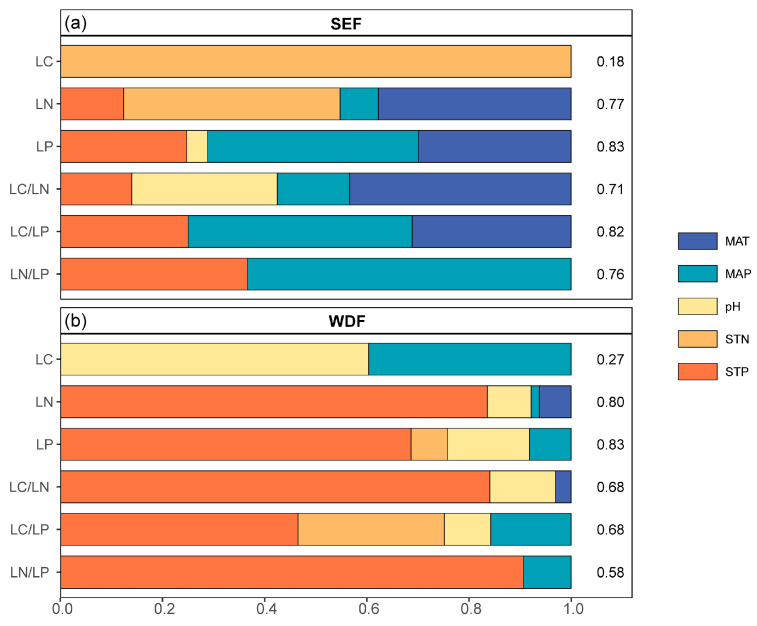
Relative contributions of environmental predictors to leaf C:N:P stoichiometric traits across the two vegetation zones. (**a**) SEF; (**b**) WDF. Relative importance was quantified using the Lindeman–Merenda–Gold (LMG) metric based on the final multiple linear regression models. In the horizontal stacked bars, different colors represent the relative contribution of each retained predictor, and the numbers on the right indicate the R^2^ of the corresponding model. Only predictors retained in the final models are shown. SEF, subtropical evergreen broad-leaved forest zone; WDF, warm-temperate deciduous broad-leaved forest zone.

The environmental associations of leaf C:N:P stoichiometric variation differed markedly between the two vegetation zones (Figure 3 and Figure 4). Variance partitioning analysis showed that, in the SEF, the independent contribution of climate factors (33.30%) was higher than that of soil factors (18.40%), with a moderate shared contribution between the two factor groups (11.10%). In the WDF, soil factors made the largest independent contribution (59.81%), whereas the contribution of climate factors was relatively weak (8.18%). The shared fraction was negative (−8.26%) and was therefore interpreted as negligible, indicating little joint explanatory contribution of climate and soil factors. Stepwise regression further indicated that most leaf stoichiometric traits in the SEF were associated with both climate and soil factors. By contrast, leaf stoichiometric variation in the WDF was better explained by soil factors, particularly STP. Overall, these results revealed distinct patterns of environmental association in leaf C:N:P stoichiometry, characterized by joint contributions of climate and soil factors in the SEF and a stronger soil-related contribution, especially from STP, in the WDF.

### 3.4. Relationships Among Leaf C:N:P Stoichiometric Traits

**Figure 5:**
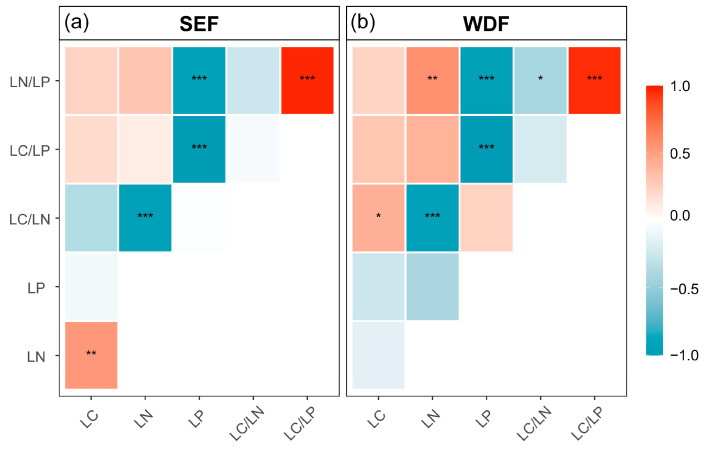
Correlations among leaf C:N:P stoichiometric traits across the two vegetation zones. (**a**) SEF; (**b**) WDF. Colors indicate Spearman’s rank correlation coefficients, with red and blue representing positive and negative correlations, respectively; darker colors indicate stronger correlations. Only the lower triangular matrix is shown. Asterisks denote significance levels: * *p* < 0.05, ** *p* < 0.01, and *** *p* < 0.001. SEF, subtropical evergreen broad-leaved forest zone; WDF, warm-temperate deciduous broad-leaved forest zone.

**Figure 6:**
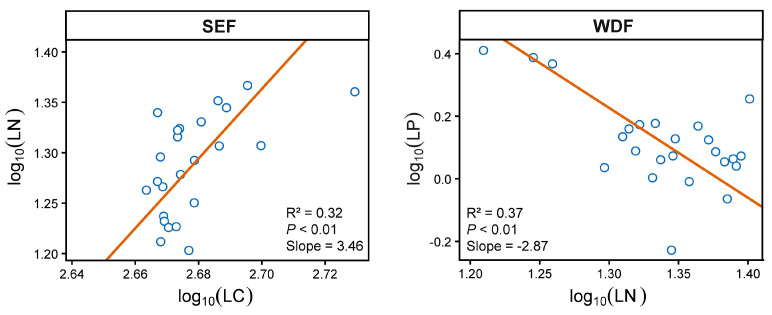
Standardized major axis relationships among leaf C, N, and P traits across the two vegetation zones. All variables were log_10_-transformed before analysis. Blue open circles represent sampling-site observations, and orange lines indicate standardized major axis (SMA) regression lines. R^2^, *p* values, and slopes are shown in each panel. Only significant SMA relationships are presented. SEF, subtropical evergreen broad-leaved forest zone; WDF, warm-temperate deciduous broad-leaved forest zone.

The relationships among leaf C:N:P stoichiometric traits differed markedly between the two vegetation zones. Overall, more significant correlations were observed in the WDF than in the SEF. In particular, LN/LP was significantly correlated with all stoichiometric traits except LC in the WDF (*p* < 0.05; Figure 5). Standardized major axis (SMA) analysis further showed different element–element relationships between the two vegetation zones (Figure 6). In the SEF, only the relationship between LC and LN was significantly positive (R^2^ = 0.32, *p* < 0.01, slope = 3.46), indicating positive covariation between leaf C and leaf N across sites. In the WDF, only the relationship between LN and LP was significantly negative (R^2^ = 0.37, *p* < 0.01, slope = −2.87), suggesting negative covariation between leaf N and leaf P across sites.

## 4. Discussion

### 4.1. Between-Zone Differentiation in Leaf C:N:P Stoichiometry

The leaf C:N:P stoichiometric traits of *Q. variabilis* differed in their magnitude of variation. LC exhibited the lowest variation in both the SEF and WDF, whereas LP and P-related stoichiometric ratios showed greater variability. Leaf C is mainly involved in leaf structural construction, and is generally constrained by structural investment, which may explain its relatively conservative variation. In contrast, leaf N and leaf P are directly involved in protein synthesis, photosynthesis, energy transfer, and metabolic regulation, and are therefore more sensitive to changes in resource supply and plant nutrient demand [1,23,24]. These contrasting patterns suggest that different leaf elements in *Q. variabilis* respond differently to environmental variation, with C-related structural investment being relatively conservative and nutrient-related elements and their stoichiometric relationships showing greater plasticity. This finding is consistent with the general patterns reported in large-scale stoichiometric studies. At both global and regional scales, leaf N, P, and N:P have been shown to exhibit pronounced geographic variation associated with temperature, latitude, and soil nutrient conditions [1,8]. A study of woody plants across eastern China further showed that leaf P was more variable and more tightly associated with climate and soil nutrient conditions than leaf N [25]. Consistent with these findings, the higher variability of LP and P-related stoichiometric ratios in *Q. variabilis* suggests that P may be an important component shaping spatial variation in leaf stoichiometry of woody plants.

Across all samples, *Q. variabilis* had mean leaf C, N, and P of 472.24, 20.75, and 1.45 g kg^−1^, respectively, and mean C:N, C:P, and N:P ratios of 23.12, 365.12, and 16.23, respectively (Table S4). Leaf N, leaf P, and the N:P ratio were broadly consistent with the mean values reported for terrestrial plants in China (N = 20.2 g kg^−1^, P = 1.45 g kg^−1^, N:P = 16.3; Ref. [8]), indicating that *Q. variabilis* generally conforms to the overall pattern of leaf stoichiometry in Chinese terrestrial plants. However, compared with the mean values reported for Chinese *Quercus* species (N = 17.27 g kg^−1^, P = 1.54 g kg^−1^, N:P = 13.96; Ref. [12]), *Q. variabilis* showed higher leaf N and N:P but lower leaf P. This suggests that, as a widely distributed species in China, *Q. variabilis* may possess nutrient characteristics that differ from the genus-level average pattern. Nevertheless, such overall consistency does not necessarily imply that *Q. variabilis* maintains the same leaf stoichiometric pattern across different vegetation zones.

Notably, significant between-zone differences were mainly detected in LN and LC/LN, rather than occurring synchronously across all leaf elements. This indicates that between-zone differentiation in *Q. variabilis* may not be achieved through a simple overall increase or decrease in leaf elemental concentrations, but may instead reflect selective adjustment in nutrient acquisition and utilization. Specifically, P-related traits contributed more strongly to overall spatial variability, whereas differentiation between vegetation zones was more evident in LN and LC/LN. This pattern may be related to differences in nutrient supply backgrounds between vegetation zones and further implies that leaf N acquisition and utilization may differ under contrasting environmental conditions [5,26].

### 4.2. Between-Zone Differences in Environmental Associations

Relationships between plant traits and environmental gradients have long been a central topic in ecology [4,27,28,29]. In this study, differences in the leaf C:N:P stoichiometry of *Q. variabilis* between the two vegetation zones may reflect zone-dependent responses to contrasting environmental constraints. Although geographic gradients, including longitude, latitude, and elevation, provide useful frameworks for examining plant trait variation, they do not directly act on plants. Instead, stoichiometric patterns along geographic gradients should be interpreted as the spatial integration of climate, soil conditions, and their associated limiting effects [7,13,30]. Previous studies have shown that geographic variation in leaf N and P is ultimately regulated by the combined effects of temperature, water availability, and soil nutrient conditions, rather than by spatial location alone [8,9,12].

Globally, leaf N and P concentrations generally decrease toward lower latitudes, whereas leaf N:P often increases toward the equator. Across China, however, leaf N and P have been reported to increase with latitude, while the latitudinal pattern of N:P is relatively weak [7,8]. In this study, leaf C:N:P stoichiometric traits in the SEF showed no significant relationships with latitude. By contrast, in the WDF, LN, LC/LP, and LN/LP increased significantly with latitude, whereas LP and LC/LN decreased significantly. This indicates that the latitudinal patterns of leaf stoichiometry of *Q. variabilis* were zone dependent. When considered in relation to the environmental background, the two vegetation zones first showed clear climatic differentiation. The SEF was characterized by warmer and wetter conditions, whereas soil conditions were broadly similar between the two zones, except for a significant difference in SBD (Tables S5 and S6). In the WDF, the latitudinal trend of LN was consistent with the general pattern reported in previous studies, whereas the trends of LP and LN/LP deviated from this expectation [8,12]. This suggests that leaf stoichiometry of *Q. variabilis* in the WDF may be associated not only with hydrothermal gradients represented by latitude, but also with local soil conditions and intraspecific adjustment to resource availability, including changes in nutrient acquisition and utilization among populations of the same species. Studies at the *Quercus* genus scale have also shown that relationships between leaf stoichiometry and geographic gradients are often more complex than those with individual climatic factors, and that hydrothermal gradients associated with latitude usually have a more direct association with leaf N than with N:P [12]. Conversely, the absence of significant latitudinal relationships in the SEF suggests that, in warm and humid subtropical regions, coupled climate–soil effects may play a stronger role in shaping leaf stoichiometry, thereby weakening the explanatory power of a single latitudinal gradient [25,31,32]. This explanation remains tentative and should be regarded as a possible interpretation rather than direct evidence.

Variance partitioning analysis and multiple regression further showed that leaf C:N:P stoichiometry was associated with both climate and soil factors in the SEF, but was mainly explained by soil factors in the WDF, particularly STP. This contrast may reflect differences in the relative explanatory roles of environmental constraints between the two vegetation zones. The SEF is generally warmer and wetter and has a longer growing season, which may influence leaf nutrient demand directly and strengthen climate–soil coupling by regulating soil nutrient transformation and availability [7,14,33]. For example, Li et al. [16] reported that the increase in leaf N:P in subtropical forests of eastern China was jointly affected by temperature, precipitation, and soil available P. By comparison, the stronger seasonality in the WDF may shorten the warm and moist period favorable for soil nutrient transformation and availability [7,14,16,33], making variation in soil nutrient supply more closely reflected in leaf stoichiometry. In addition, subtropical and warm-temperate forests may differ in their background of relative nutrient limitation. Previous studies have shown that subtropical forests are more likely to exhibit P limitation, whereas warm-temperate forests may be more prone to N limitation or N–P co-limitation [16,34,35]. These results indicate differences in the relative contributions of climate and soil variables between the two vegetation zones, rather than contradictory effects of climate and soil factors. Regional-scale nutrient limitation provides the broader environmental context for interpreting among-site stoichiometric variation within each vegetation zone.

### 4.3. Elemental Coordination and Trade-Offs

Building on the observed between-zone differentiation and contrasting environmental associations, the relationships among leaf elements in *Q. variabilis* also differed between vegetation zones. This suggests that between-zone differences in leaf stoichiometry were not limited to changes in individual elements, but also involved shifts in element–element relationships. Leaf C primarily forms the structural basis of leaves, leaf N is closely associated with protein synthesis and photosynthetic enzyme systems, and leaf P is involved in nucleic acid synthesis, energy transfer, and growth regulation. Thus, the relationships among leaf C, N, and P broadly reflect how plants coordinate carbon investment and nutrient use under specific environmental contexts [1,5].

From the perspective of specific element–element relationships, the SEF was mainly characterized by a positive C–N relationship, whereas the WDF showed a negative N–P relationship. In addition, LC was relatively weakly associated with other stoichiometric traits, whereas LN, LP, and their related ratios were more tightly linked. This further indicates that the differences between the two vegetation zones were not limited to changes in elemental concentrations, but also reflected differences in how leaf elements covaried. The positive C–N relationship in the SEF suggests that leaf structural construction and N-related metabolic investment were more closely linked under warmer and wetter conditions, where leaf stoichiometry was associated with both climate and soil factors. Warmer and wetter conditions may support higher metabolic activity and promote closer coupling between C-based structural construction and N-related investment in photosynthetic proteins and enzyme systems [6,36,37]. This may explain the positive covariation between leaf C and leaf N in the SEF. By contrast, the negative N–P relationship in the WDF suggests that, under stronger seasonality and more direct soil-related background constraints in the warm-temperate zone, plants may show different adjustments in N and P use [5,38,39]. In this study, leaf stoichiometric variation in the WDF was mainly explained by soil factors, particularly STP, indicating that the N–P relationship in this zone more likely reflects adjustment in nutrient acquisition and use under soil resource constraints, rather than simple synchronous accumulation of elements.

From the perspective of allometric relationships, previous studies have shown that leaf N and P often follow a relatively stable power-law scaling relationship, with a scaling exponent close to 2/3 at broad spatial scales [4,40]. However, this relationship is not strictly invariant. Based on a global analysis of leaf N–P relationships, Tian et al. [41] found that scaling exponents varied across different plant functional groups, latitudinal regions, and ecoregions. Similarly, Duan et al. [9] further showed that leaf N–P scaling exponents in Chinese woody plants varied with life form and environmental background. These findings indicate that there is no universally fixed scaling exponent for leaf N–P relationships. Therefore, the absence of a broad-scale positive N–P scaling pattern in *Q. variabilis* does not necessarily indicate a deviation from the general pattern. Rather, it may reflect the zone-dependent responses of a single widely distributed tree species to contrasting environmental conditions across vegetation zones.

**Figure 7:**
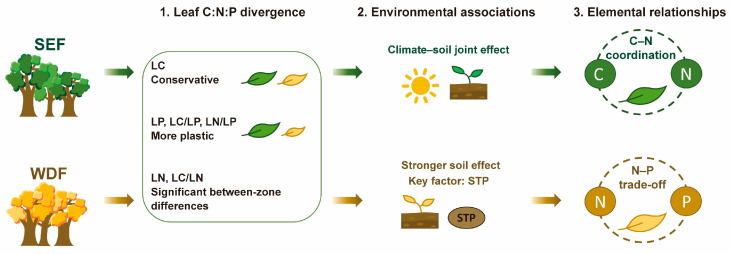
Conceptual framework linking leaf C:N:P stoichiometry, contrasting environmental associations, and elemental relationships in *Q. variabilis*. The framework illustrates zone-dependent stoichiometric adjustment across the SEF and WDF. LC was relatively conservative, whereas LP and P-related stoichiometric ratios were more plastic, and significant between-zone differences were mainly concentrated in LN and LC/LN. The SEF was characterized by a climate–soil joint effect and C–N coordination, whereas the WDF showed a stronger soil effect, with STP as a key factor, and a more pronounced N–P trade-off. Arrows indicate the conceptual links among stoichiometric divergence, environmental associations, and elemental relationships. SEF, subtropical evergreen broad-leaved forest zone; WDF, warm-temperate deciduous broad-leaved forest zone.

Overall, the differentiation of leaf C:N:P stoichiometry in *Q. variabilis* across vegetation zones in China reveals a zone-dependent framework of stoichiometric adjustment (Figure 7). At the elemental level, LC was relatively conservative, whereas LP and P-related stoichiometric ratios were more plastic, indicating that spatial variation was mainly concentrated in nutrient-related elements and their stoichiometric relationships. In terms of environmental associations, the warmer and wetter conditions in the SEF may strengthen climate–soil coupling, leading to joint climate–soil contributions to leaf stoichiometry. By comparison, stronger seasonality in the WDF may increase the explanatory role of soil factors, particularly STP. At the level of elemental relationships, C–N coordination in the SEF reflected enhanced coupling between structural construction and metabolic investment, whereas the WDF showed a more pronounced N–P trade-off, suggesting different adjustments in N and P use under stronger soil constraints. Taken together, *Q. variabilis* does not appear to rely on a single fixed nutrient-use strategy across its broad distribution. Instead, it may maintain leaf function and ecological adaptation under contrasting environmental constraints through zone-dependent stoichiometric adjustment.

## 5. Conclusions

This study shows that the leaf C:N:P stoichiometry of *Q. variabilis* exhibits between-zone differentiation across vegetation zones in China, mainly reflected in LN and LC/LN. Overall, LC was relatively conservative, whereas LP and P-related stoichiometric ratios showed greater plasticity. Compared with the SEF, the WDF showed stronger associations with geographic gradients, especially the latitudinal gradient. The two vegetation zones also differed markedly in environmental associations and elemental relationships. Leaf stoichiometry in the SEF was associated with joint contributions of climate and soil factors and was characterized by C–N coordination, whereas that in the WDF was mainly explained by soil factors, particularly STP, and showed a more pronounced N–P trade-off. Overall, these findings largely support our initial hypothesis that *Q. variabilis* exhibits zone-dependent stoichiometric adjustment rather than a fixed leaf stoichiometric pattern across vegetation zones. These findings also highlight the importance of vegetation-zone context in understanding intraspecific variation in leaf C:N:P stoichiometry of widely distributed tree species.

## Figures and Tables

**Table 1:** Basic information on the sampling sites.

Site	Zone	Longitude (°E)	Latitude (°N)	Altitude (m)	MAT (°C)	MAP (mm)	SBD (g cm^−3^)	pH	SOC (%)	STN (g kg^−1^)	STP (mg kg^−1^)
Pinggu, Beijing	WDF	117.12	40.25	222.25	12	882	1.71	8.0	1.05	1.02	681.37
Fengjie, Chongqing	SEF	109.14	31.29	622.47	17.7	1947	1.85	5.1	3.03	2.25	539.70
Tianshui, Gansu	WDF	106.65	34.32	1029.43	11.8	950	1.71	8.0	1.05	1.02	583.61
Tianlin, Guangxi	SEF	105.92	24.44	556.00	21.1	2496	1.78	6.3	1.44	1.40	584.30
Daozhen, Guizhou	SEF	107.47	29.01	1036.76	16.2	1574	1.59	6.2	2.57	1.91	503.63
Xingtai, Hebei	WDF	113.88	37.11	530.25	14.6	1116	1.66	5.9	1.51	1.33	698.14
Yixian, Hebei	WDF	115.48	39.48	506.88	12.5	1180	1.71	5.2	2.64	2.32	711.02
Lushi, Henan	WDF	110.98	33.94	687.06	14.4	1025	1.66	5.9	1.51	1.33	565.75
Xinyang, Henan	SEF	114.04	32.11	189.46	15.9	2149	1.72	6.1	0.97	0.79	572.24
Yongxiu, Jiangxi	SEF	115.63	29.08	201.15	17.9	2795	1.78	6.3	1.44	1.40	665.43
Jinan, Shandong	WDF	117.08	36.34	609.75	6.2	1901	1.77	7.9	0.91	0.89	630.80
Huanglong, Shaanxi	WDF	110.11	35.54	904.29	9.4	781	1.71	6.4	1.56	1.41	689.73
Zhouzhi, Shaanxi	WDF	108.03	34.13	580.00	13.8	1094	1.70	5.2	1.33	1.13	679.53
Zhongjiang, Sichuan	SEF	104.60	31.01	742.31	17.1	1774	1.78	6.3	1.44	1.40	555.25
Kunming, Yunnan	SEF	102.62	24.97	2136.57	16	1648	1.77	7.9	0.91	0.89	626.12
Hangzhou, Zhejiang	SEF	120.01	30.19	254.43	17.5	1794	1.90	6.1	1.93	1.32	790.73

MAT, mean annual temperature; MAP, mean annual precipitation; SBD, soil bulk density; SOC, soil organic carbon; STN, soil total nitrogen; STP, soil total phosphorus. Soil variables refer to the 0–20 cm soil layer. Variables in Table S1 are presented in their original units as obtained from the source datasets. SEF, subtropical evergreen broad-leaved forest zone; WDF, warm-temperate deciduous broad-leaved forest zone. SEF = 8 sites, WDF = 8 sites, total = 16 sites.

**Table 2:** Descriptive statistics of leaf C:N:P stoichiometry across the two vegetation zones.

Zone	Trait	Mean	SD	Min	Max	CV (%)
SEF	LC (g kg^−1^)	477.05 a	16.31	460.78	536.27	3.42
	LN (g kg^−1^)	19.55 b	2.21	15.96	23.27	11.32
	LP (g kg^−1^)	1.52 a	0.51	0.75	2.43	33.28
	LC/LN	24.66 a	2.48	21.24	29.77	10.07
	LC/LP	352.68 a	130.13	191.43	617.74	36.90
	LN/LP	14.49 a	5.78	8.13	26.07	39.93
WDF	LC (g kg^−1^)	467.44 a	14.63	439.01	497.39	3.13
	LN (g kg^−1^)	21.95 a	2.37	16.20	25.19	10.79
	LP (g kg^−1^)	1.37 a	0.48	0.59	2.57	35.00
	LC/LN	21.58 b	2.86	17.82	29.58	13.26
	LC/LP	377.55 a	129.42	186.19	801.38	34.28
	LN/LP	17.97 a	6.61	6.30	37.41	36.79

SEF, subtropical evergreen broad-leaved forest zone; WDF, warm-temperate deciduous broad-leaved forest zone. LC, leaf carbon; LN, leaf nitrogen; LP, leaf phosphorus; SD, standard deviation; Min, minimum; Max, maximum; CV, coefficient of variation. Different lowercase letters within the same trait indicate significant differences between vegetation zones at *p* < 0.05.

## Data Availability

The datasets generated or analyzed during the current study are available from the corresponding author upon reasonable request.

## Supplementary Materials

The following supporting information can be downloaded at: https://www.mdpi.com/article/10.3390/plants15142219/s1, Table S1: Variance partitioning of climate and soil contributions to leaf C:N:P stoichiometric variation across the two vegetation zones; Table S2: Final multiple linear regression models for leaf C:N:P stoichiometric traits across the two vegetation zones; Table S3: Spearman correlation coefficients among leaf C:N:P stoichiometric traits across the two vegetation zones; Table S4: Overall descriptive statistics of leaf C:N:P stoichiometric traits across all samples; Table S5: Descriptive statistics of climate and soil variables across the two vegetation zones; Table S6: Wilcoxon rank-sum test results of environmental variables between the two vegetation zones.
