# Supplementary material for: Climate and Soil Constraints Shape Vegetation-Zone-Dependent Leaf C:N:P Stoichiometry of Quercus variabilis
Source: Plants (Basel). 2026 Jul 21;15(14):2219. doi: 10.3390/plants15142219 (PMC13417380; doi:10.3390/plants15142219)
Supplement: Supplementary file 1 [file plants-15-02219-s001.zip › plants-4401837-supplementary.pdf]

Supplementary Material

Table S1. Variance partitioning of climate and soil contributions to leaf C:N:P stoichiometric variation across the two vegetation zones.

Zone	Component	Adj R^2	F	*P*
SEF	Climate	0.3330	9.9512	0.001
	Soil	0.1840	4.4614	0.001
	Shared	0.1110	—	—
	Residual	0.3720	—	—
	Total	0.6280	8.7646	0.001
WDF	Climate	0.0818	3.0319	0.009
	Soil	0.5981	11.3980	0.001
	Shared	−0.0826	—	—
	Residual	0.4027	—	—
	Total	0.5973	7.8239	0.001

Adjusted R^2 represents the adjusted coefficient of determination. Climate and Soil denote the independent fractions explained by climate and soil variables, respectively; Shared represents the fraction jointly explained by both variable groups; and Residual indicates unexplained variation. Total refers to the overall explained variation. Significance was tested using RDA and partial RDA. The shared fraction was not tested for significance. The negative shared value in the WDF resulted from adjusted R^2 estimation and was interpreted as negligible rather than as a meaningful negative joint effect. SEF, subtropical evergreen broad-leaved forest zone; WDF, warm-temperate deciduous broad-leaved forest zone.

Table S2. Final multiple linear regression models for leaf C:N:P stoichiometric traits across the two vegetation zones.

Zone	Trait	Formula	R^2	Adj R^2	AIC	Model *P*
SEF	LC	LC ~ STN	0.1831	0.1460	202.2256	0.0370
SEF	LN	LN ~ STN + MAT + MAP + STP	0.7685	0.7198	82.0914	0.0000
SEF	LP	LP ~ MAP + STP + MAT + pH	0.8298	0.7940	3.9923	0.0000
SEF	LC/LN	LC/LN ~ MAT + MAP + STP + pH	0.7118	0.6511	92.8729	0.0001
SEF	LC/LP	LC/LP ~ MAP + STP + MAT	0.8155	0.7878	270.2134	0.0000
SEF	LN/LP	LN/LP ~ MAP + STP	0.7633	0.7407	124.7555	0.0000
WDF	LC	LC ~ pH + MAP	0.2696	0.2001	196.3212	0.0369
WDF	LN	LN ~ STP + MAT + pH + MAP	0.7952	0.7521	82.3872	0.0000
WDF	LP	LP ~ STP + pH + MAP + STN	0.8300	0.7943	1.4184	0.0000
WDF	LC/LN	LC/LN ~ STP + pH + MAT	0.6848	0.6375	99.8319	0.0000
WDF	LC/LP	LC/LP ~ STP + MAP + STN + pH	0.6768	0.6087	285.4117	0.0002
WDF	LN/LP	LN/LP ~ STP + MAP	0.5832	0.5435	144.7496	0.0001

The table summarizes the final multiple linear regression models for leaf C:N:P stoichiometric traits across the SEF and WDF, including model formula, coefficient of determination (R^2), adjusted coefficient of determination (Adj. R^2), Akaike information criterion (AIC), and overall model significance. Final models were selected using bidirectional stepwise model selection. SEF, subtropical evergreen broad-leaved forest zone; WDF, warm-temperate deciduous broad-leaved forest zone.

Table S3. Spearman correlation coefficients among leaf C:N:P stoichiometric traits across the two vegetation zones.

Zone	Trait	LC	LN	LP	LC/LN	LC/LP	LN/LP

SEF	LC	1.0000	0.5287	−0.0722	−0.3626	0.1922	0.2330
	LN	0.5287	1.0000	−0.0078	−0.9600	0.1061	0.2991
	LP	−0.0722	−0.0078	1.0000	−0.0183	−0.9826	−0.9339
	LC/LN	−0.3626	−0.9600	−0.0183	1.0000	−0.0443	−0.2470
	LC/LP	0.1922	0.1061	−0.9826	−0.0443	1.0000	0.9652
	LN/LP	0.2330	0.2991	−0.9339	−0.2470	0.9652	1.0000
WDF	LC	1.0000	−0.1331	−0.2496	0.4175	0.3035	0.2292
	LN	−0.1331	1.0000	−0.4043	−0.9365	0.3922	0.5722
	LP	−0.2496	−0.4043	1.0000	0.2383	−0.9904	−0.9574
	LC/LN	0.4175	−0.9365	0.2383	1.0000	−0.2009	−0.4165
	LC/LP	0.3035	0.3922	−0.9904	−0.2009	1.0000	0.9487
	LN/LP	0.2292	0.5722	−0.9574	−0.4165	0.9487	1.0000

Values represent Spearman's rank correlation coefficients. Negative values are indicated by minus signs. SEF, subtropical evergreen broad-leaved forest zone; WDF, warm-temperate deciduous broad-leaved forest zone.

Table S4. Overall descriptive statistics of leaf C:N:P stoichiometric traits across all samples.

Trait	Mean	SD	Min	Max	CV (%)
LC (g kg^{-1})	472.24	16.07	439.01	536.27	3.40%
LN (g kg^{-1})	20.75	2.57	15.96	25.19	12.39%
LP (g kg^{-1})	1.45	0.49	0.59	2.57	34.15%
LC/LN	23.12	3.07	17.82	29.77	13.29%
LC/LP	365.12	129.00	186.19	801.38	35.33%
LN/LP	16.23	6.39	6.30	37.41	39.40%

LC, leaf carbon; LN, leaf nitrogen; LP, leaf phosphorus; SD, standard deviation; Min, minimum; Max, maximum; CV, coefficient of variation.

Table S5. Descriptive statistics of climate and soil variables across the two vegetation zones.

Zone	Trait	Mean	SD	Min	Max	CV (%)
SEF	MAP (mm)	2022.13	430.62	1574.00	2795.00	21.30
	MAT (°C)	17.43	1.68	15.90	21.10	9.65
	pH	6.29	0.76	5.10	7.90	12.16
	SBD (g cm^{-3})	1.77	0.09	1.59	1.90	5.16
	SOC (g kg^{-1})	17.15	7.51	9.07	30.29	43.81
	STN (g kg^{-1})	1.42	0.48	0.79	2.25	33.86
	STP (g kg^{-1})	0.60	0.09	0.50	0.79	14.94
WDF	MAP (mm)	1116.13	343.02	781.00	1901.00	30.73
	MAT (°C)	11.84	2.83	6.20	14.60	23.94
	pH	6.56	1.23	5.20	8.00	18.70
	SBD (g cm^{-3})	1.70	0.03	1.66	1.77	2.03
	SOC (g kg^{-1})	14.46	5.44	9.07	26.44	37.59
	STN (g kg^{-1})	1.31	0.45	0.89	2.32	34.33
	STP (g kg^{-1})	0.65	0.05	0.57	0.71	8.39

MAT, mean annual temperature (°C); MAP, mean annual precipitation (mm); SBD, soil bulk density (g cm^{-3}); SOC, soil organic carbon (g kg^{-1}); STN, soil total nitrogen (g kg^{-1}); STP, soil total phosphorus (g kg^{-1}). Soil variables refer to the 0–20 cm soil layer and were converted to consistent units before statistical analysis. SEF, subtropical evergreen broad-leaved forest zone; WDF, warm-temperate deciduous broad-leaved forest zone.

Table S6. Wilcoxon rank-sum test results of environmental variables between the two vegetation zones.

\	W	Adj *P*	Significance
MAP	60.0	0.0050	**
MAT	64.0	0.0006	***

pH	29.5	0.8330	ns
SBD	54.5	0.0388	*
SOC	35.5	0.8330	ns
STN	36.5	0.8330	ns
STP	16.0	0.1680	ns

W is the Wilcoxon rank-sum test statistic, and Adj *P* is the Holm-adjusted *P* value. Significance levels are denoted by ns, *, **, and ***, corresponding to not significant, $P < 0.05$, $P < 0.01$, and $P < 0.001$, respectively.
